# Willingness of Pharmacists to Prescribe Medication Abortion in California

**DOI:** 10.1001/jamanetworkopen.2024.6018

**Published:** 2024-04-10

**Authors:** Cathren Cohen, Lauren A. Hunter, Raiza M. Beltran, Jaclyn Serpico, Laura Packel, Ayako Miyashita Ochoa, Sandra I. McCoy, Kerith J. Conron

**Affiliations:** 1 Center on Reproductive Health, Law, and Policy, UCLA (University of California, Los Angeles) School of Law; 2 School of Public Health, University of California, Berkeley; 3 Luskin School of Public Affairs, UCLA; 4 Now with School of Public Health, University of Minnesota, Minneapolis; 5 Now with Malkia Klabu Program, University of California, San Francisco; 6 Williams Institute, UCLA School of Law

## Abstract

**Importance:**

Nearly half of US states have restricted abortion access. Policy makers are exploring pathways to increase access to abortion and reproductive health care more broadly. Since 2016, California pharmacists could prescribe hormonal birth control, providing an opportunity to learn about the implementation of pharmacist-provided reproductive health care.

**Objective:**

To explore the feasibility of broadening pharmacist scope of practice to include prescribing medication abortion.

**Design, Setting, and Participants:**

A cross-sectional online survey was conducted from October 11 to December 20, 2022, among a convenience sample of California licensed community pharmacists to examine their attitudes toward, knowledge of, and confidence in prescribing hormonal birth control and reports of pharmacy-level practices.

**Main Outcomes and Measures:**

Descriptive analyses and log-binomial regression models were used to compare medication abortion and contraceptive provision attitudes by pharmacist and pharmacy characteristics.

**Results:**

Among the 316 pharmacists included in the analysis who worked at community pharmacies across California (mean [SD] age, 40.9 [12.0] years; 169 of 285 [59.3%] cisgender women; and 159 of 272 [58.5%] non-Hispanic Asian individuals), most (193 of 280 [68.9%]) indicated willingness to prescribe medication abortion to pharmacy clients if allowed by law. However, less than half were confident in their knowledge of medication abortion (139 of 288 [48.3%]) or their ability to prescribe it (115 of 285 [40.4%]). Pharmacists who indicated that providing access to hormonal birth control as a prescribing provider was important (263 of 289 [91.0%]) and were confident in their ability to prescribe it (207 of 290 [71.4%]) were 3.96 (95% CI, 1.80-8.73) times and 2.44 (95% CI, 1.56-3.82) times more likely to be willing to prescribe medication abortion and to express confidence in doing so, respectively. Although most pharmacists held favorable attitudes toward hormonal birth control, less than half (144 of 308 [46.8%]) worked in a pharmacy that provided prescriptions for hormonal birth control, and 149 who did not reported barriers such as lack of knowledge or training (65 [43.6%]), insufficient staff or time to add new services (58 [38.9%]), and lack of coverage for services (50 [33.6%]).

**Conclusions and Relevance:**

The findings of this cross-sectional survey study of California pharmacists suggest that most pharmacists were willing to prescribe medication abortion. However, future efforts to expand pharmacists’ scope of practice should include training to increase knowledge and confidence in prescribing medication abortion. Pharmacy-level barriers to hormonal birth control prescription, such as insurance coverage for pharmacist effort, should also be addressed, as they may serve as barriers to medication abortion access.

## Key Points

**Question:**

Are pharmacists in California willing to prescribe medication abortion?

**Findings:**

In this cross-sectional survey study of 316 California community pharmacists, 193 of 280 (69%) were willing to prescribe medication abortion if permitted by law, but only 139 of 288 (48%) were confident in their knowledge and 115 of 285 (40%) were confident in their ability to do so. Despite greater willingness and confidence to prescribe hormonal birth control, only 144 of 308 pharmacists (47%) worked in pharmacies that provided these prescriptions; those who worked at pharmacies that did not provide these prescriptions reported knowledge or training, staffing or time, and payment for services as barriers.

**Meaning:**

These findings suggest that most pharmacists in California would be willing to prescribe medication abortion if legally permitted to do so; however, training and attention to pharmacy-level barriers may be needed.

## Teaser

This cross-sectional study surveys attitudes toward reproductive health services and medication abortion; the availability of pharmacist-prescribed, self-administered hormonal contraceptives; and pharmacy-level contraceptive implementation obstacles from licensed community pharmacists in California.

## Introduction

Following the US Supreme Court’s decision in Dobbs v Jackson Women’s Health, half of states have banned or severely restricted abortion care or are expected to do so.^1^ As a result, clinicians in states where abortion remains legal are facing increased demand for services.^2,3^ Policy makers are exploring how to increase access to abortion services and reproductive health care more broadly, while also contending with COVID-19–induced clinician burnout and workforce shortages.^4,5^

Medication abortion accounts for more than half (54%) of all abortions in the US^6^ and consists of a regimen of 2 medications—mifepristone and misoprostol—taken within days of each other.^7^ Mifepristone has historically been subject to strict regulation under the US Food and Drug Administration Risk Evaluation and Mitigation Strategies program, which regulates how and where the drug can be dispensed, and by whom.^8^ These federal regulations on mifepristone recently changed to remove a prior Risk Evaluation and Mitigation Strategies requirement that mifepristone be dispensed in person by a certified prescriber and to allow pharmacists at certified retail pharmacies to dispense mifepristone.^9^ Provision of medication abortion in pharmacies thus represents an opportunity to increase access to abortion care and reduce the burden on the health care system. Although multiple lawsuits implicating the legal status of mifepristone and the conditions under which it can be dispensed have been filed across the country, an emergency order from the Supreme Court keeps regulations governing mifepristone unchanged while litigation is ongoing.^10^

Both the American Medical Association and American College of Obstetricians and Gynecologists have expressed support for pharmacists dispensing medication abortion,^11,12^ and pilot programs have shown that pharmacists can safely and effectively do so.^13,14^ In these pilot programs, patients who received pharmacist-provided abortion medication and follow-up care reported having satisfactory abortion experiences.^13,14^ Additionally, previous studies conducted in the US and Canada,^14,15,16,17,18,19,20^ where dispensing of medication abortion by pharmacists has been legal since 2017, demonstrate pharmacists’ willingness to dispense medication abortion. Among US-based pharmacists, benefits to dispensing medication abortion include the ability to expand abortion access to patients, improve patients’ quality of care, streamline delivery of health care services, and make use of pharmacists’ expertise.^14,15^ Pharmacists also identified potential barriers to medication abortion dispensing such as employer hesitancy, a lack of private space for patient consultations, safe follow-up for postabortion care, adequate staffing and training needs, established reimbursement mechanisms for medication abortion–related services, and having colleagues with religious, political, or personal objections to providing medication abortion.^14,16,20^ Studies conducted in Canada found similar barriers to dispensing medication abortion among pharmacists, with the addition of having low demand among patients, drug shortages, and short expiration dates.^17,18,19^ Indeed, more information is needed to inform public health efforts to expand the provision of medication abortion. Twenty-seven states, including California, currently allow pharmacists to prescribe hormonal contraceptives, affording a unique opportunity to learn about this pathway to reproductive health services.^21^

This survey project, the California Pharmacist Study, gathered information about attitudes toward reproductive health services and medication abortion, the availability of pharmacist-prescribed self-administered hormonal contraceptives, and pharmacy-level contraceptive implementation obstacles from licensed community pharmacists. We hypothesized that pharmacists who held favorable attitudes and practices toward pharmacist-provided hormonal birth control would be more likely to hold favorable attitudes toward medication abortion. Information about implementation experiences with pharmacist prescription of hormonal birth control, which has been legal in California for nearly a decade, is presented and discussed relative to the potential to include medication abortion under an expanded scope of practice.

## Methods

### Study Design and Sample

The cross-sectional California Pharmacist Study survey was conducted between October 11 and December 20, 2022, with a convenience sample of pharmacists and pharmacy students 18 years and older who reside in the State of California. A target sample size of 1000 was selected to enable comparisons of sexual and reproductive health service availability and training needs across regions of the state (ie, Los Angeles County, Greater Bay Area, other urban areas, and rural areas) with differing health service landscapes. A multistage recruitment plan included both online and in-person recruitment. In the first phase, participants were recruited through the California Society of Health-System Pharmacists and California Pharmacists Association membership email listservs and newsletters. Information about the study was distributed through flyers and presentations at the annual meetings of the American College of Clinical Pharmacy and the California Society of Health-System Pharmacists. During the second phase of recruitment, the survey link was shared on the social media channels of the California Society of Health-System Pharmacists and California Pharmacists Association and professional groups representing pharmacists in specific regions (eg, California’s rural Central Valley) and pharmacists of specific racial and ethnic identities (eg, Black or African American pharmacists).

Approval for this study was granted by the Office of the Human Research Protection Program Institutional Review Board at UCLA, with partner organizations holding reliance agreements. All participants indicated their consent to participate in Qualtrics after reviewing an information sheet and before initiating the survey; a waiver of written consent was obtained for study. This study followed the American Association for Public Opinion Research (AAPOR) reporting guidance for survey studies.

### Survey Instrument

The survey was developed through an iterative process that included drafting by a core multidisciplinary team, feedback from the larger project team of pharmacist researchers and students, revision, and final review and edits to ensure question clarity and relevance to pharmacy practice and policy. Survey modules included demographic information (eg, self-reported age, sex assigned at birth, gender, race, Hispanic ethnicity); professional information (years of experience, training, whether currently practicing); knowledge, attitudes, and confidence in pharmacist prescribing of hormonal contraception, emergency contraception, and medication abortion; and pharmacy information (availability of pharmacist-prescribed reproductive health resources, implementation barriers, client characteristics).

Questions about sex assigned at birth and gender were used to classify respondents as cisgender women, cisgender men, and gender-fluid or nonbinary individuals; those who provided concordant responses to sex assigned at birth and gender were considered cisgender. Race options (select all that apply) included American Indian or Alaska Native, Asian, Black or African American, Native Hawaiian or other Pacific Islander, White, other (specify), or prefer not to state. Participants were also asked if they identify as Hispanic or Latino. Participants were first classified as Hispanic or non-Hispanic. Non-Hispanic respondents were then categorized by single race selected or as multiracial. The demographic characteristics of pharmacists, including but not limited to race and ethnicity, may be correlated with attitudes toward reproductive health care and the demographic composition of pharmacy catchment areas and thus are relevant to understanding access to care.

### Survey Procedure

After providing informed consent, participants completed a self-administered online Qualtrics survey. After completing the survey, participants had the option to enter their email address to receive a $20 gift card and/or enter weekly ($250) or grand prize ($500) raffles. Given that surveys administered online with monetary incentives are a common target of bot attacks,^22^ automated fraud detection features offered by Qualtrics were used to identify and later exclude duplicate responses and bots. Only participants verified as valid following data cleaning procedures were included in the sample and were eligible for gift cards and raffle prizes.

### Statistical Analysis

Analyses were restricted to participants who reported being licensed and were currently or most recently working in a community pharmacy. This group of pharmacists was able to report on the availability of reproductive health resources and services in settings that are widely accessible to the public and represent those who can serve as a vehicle for distribution to the public (vs those who work, for example, in hospitals, mail order, or home care or who are in training and have yet to select an employment setting). However, to understand the broader views of this community of professionals, we also examined attitudes around contraception and medication abortion provision among all surveyed pharmacists and pharmacy students.

Descriptive analyses, including proportions (excluding missing and not applicable responses) with binomial or multinomial 95% CIs, were estimated. Log-binomial regression models were used to generate unadjusted prevalence ratios (PRs) comparing (1) participants’ report of whether their pharmacy provides self-administered hormonal contraception without an outside clinician’s prescription and (2) participants’ attitudes around medication abortion provision, by participant and pharmacy characteristics. All analyses were conducted in R, version 4.2.1 (R Project for Statistical Computing).

## Results

Out of the full sample of 919 participants, 316 reported being licensed pharmacists and were currently or most recently working in a community pharmacy. Of these 316 participants, the mean (SD) age was 40.9 (12.0) years (eTable 1 in Supplement 1). Among the 285 participants with available information, 169 (59.3% [95% CI, 53.7%-65.4%]) were cisgender women, 114 (40.0% [95% CI, 34.4%-46.1%]) were cisgender men, and 2 (0.7% [95% CI, 0.0-6.8%]) were gender-fluid or nonbinary. Among the 272 participants with race and ethnicity information available, 159 (58.5% [95% CI, 52.6%-64.6%]) were non-Hispanic Asian, 84 (30.9% [95% CI, 25.0%-37.0%] were non-Hispanic White, and 29 (10.7% [95% CI, 4.8%-16.8%]) were of other race or ethnicity (including Black or African American, Hispanic or Latino, Native Hawaiian or other Pacific Islander, multiracial, or other) (Table). Participants were demographically similar to California pharmacists (eTable 1 in Supplement 1). One hundred twenty-five of 296 participants (42.2% [95% CI, 36.5%-48.0%]) indicated that they could provide services in at least 1 language other than English (Table). Over half of participants (176 [55.7% (95% CI, 50.3%-61.6%)]) worked at chain pharmacies and 131 (41.5% [95% CI, 36.1%-47.4%]) worked at independent pharmacies in regions across California. Nearly half (144 of 296 [48.6% (95% CI, 42.9%-54.9%)]) worked at pharmacies where Medi-Cal was the primary insurance held by most clients.

**Table.** Characteristics of Practicing Licensed Pharmacists and Community Pharmacies in the California Pharmacist Survey, 2022

Characteristic	No. (%) [95% CI] (N = 316)^a^
**Participants**	
Age, y	
20-34	98 (33.1) [27.0-39.5]
35-44	104 (35.1) [29.1-41.5]
≥45	94 (31.8) [25.7-38.2]
Gender	
Cisgender woman	169 (59.3) [53.7-65.4]
Cisgender man	114 (40.0) [34.4-46.1]
Gender-fluid or nonbinary	2 (0.7) [0.0-6.8]
Race and ethnicity	
Non-Hispanic Asian	159 (58.5) [52.6-64.6]
Non-Hispanic White	84 (30.9) [25.0-37.0]
Other^b^	29 (10.7) [4.8-16.8]
Proficient language(s) for service provision	
English only	171 (57.8) [52.0-63.6]
≥1 Other language	125 (42.2) [36.5-48.0]
Proficient language(s) for service provision^c^	
English	280 (94.6) [92.6-97.2]
Chinese	41 (13.9) [10.1-17.6]
Spanish	37 (12.5) [9.1-16.3]
Vietnamese	23 (7.8) [5.1-10.7]
Other	37 (12.5) [9.1-16.3]
**Participants’ pharmacies**	
Pharmacy type	
Chain	176 (55.7) [50.3-61.6]
Independent	131 (41.5) [36.1-47.4]
None of the above	9 (2.8) [0.0-8.8]
Pharmacy region	
Los Angeles County	104 (33.3) [27.9-39.4]
San Francisco Bay Area	60 (19.2) [13.8-25.3]
Orange County	35 (11.2) [5.8-17.3]
Superior California^d^	26 (8.3) [2.9-14.4]
Other	87 (27.9) [22.4-34.0]
Type of insurance held by most clients	
Private insurance	102 (34.5) [28.7-40.7]
Medi-Cal or Medicaid	144 (48.6) [42.9-54.9]
Medicare	43 (14.5) [8.8-20.8]
Uninsured or other insurance	7 (2.4) [0.0-8.6]
Pharmacy provides self-administered hormonal contraception without an outside provider’s prescription	
Yes	144 (46.8) [41.2-52.9]
No	149 (48.4) [42.9-54.5]
Not sure or do not know	15 (4.9) [0.0-11.0]
Pharmacy provides levonorgestrel emergency contraception without an outside provider’s prescription	
Yes	243 (78.9) [74.7-83.5]
No	53 (17.2) [13.0-21.8]
Not sure or do not know	12 (3.9) [0.0-8.5]

^a^ Missing responses (excluded from percentages) totaled 20 for age, 31 for gender, 44 for race and ethnicity, 20 for language, 4 for pharmacy region, 20 for type of insurance, and 8 for contraceptive provision.

^b^ Includes Black or African American, Hispanic or Latino, Native Hawaiian or Other Pacific Islander, multiracial, or other.

^c^ Categories are not mutually exclusive.

^d^ Consists of Sacramento County and 16 other Northern California counties per 2020 Census Region definition.

### Hormonal Birth Control and Emergency Contraception

**Figure 1.**
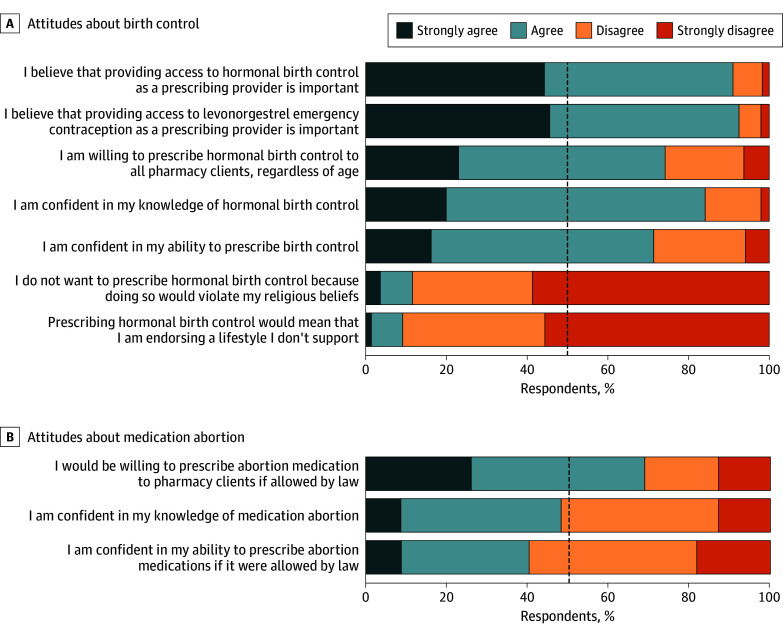
Attitudes About Birth Control and Medication Abortion Provision in the California Pharmacist Survey (n = 316), 2022 Responses exclude missing and not applicable responses (see eTable 2 in Supplement 1).

As shown in Figure 1, the community pharmacists surveyed in this project held favorable attitudes toward pharmacist-provided birth control, including hormonal birth control (263 of 289 [91.0% (95% CI, 87.1%-94.0%)] in favor) and emergency contraception (272 of 294 [92.5% (95% CI, 88.9%-95.3%)] in favor) (eTable 2 in Supplement 1). Slightly fewer participants reported confidence in their knowledge of hormonal birth control (249 of 296 [84.1% (95% CI, 79.5%-88.1%)]) and ability to prescribe birth control (207 of 290 [71.4% (95% CI, 65.8%-76.5%)]) than agreed that providing access to hormonal birth control as a prescribing provider was important (91.0%). Most participants (213 of 287 [74.2% (95% CI, 68.7%-79.2%)]) indicated their willingness to prescribe hormonal birth control to all pharmacy clients, regardless of age (in keeping with California law). Few participants indicated that prescribing birth control would violate their religious beliefs (32 of 276 [11.6% (95% CI, 8.1%-16.0%)]) or would mean that they are endorsing a lifestyle they do not support (26 of 284 [9.2% (95% CI, 6.1%-13.1%)]). Findings were similar in the broader sample of 919 pharmacists (eTable 3 in Supplement 1).

Slightly less than half of pharmacists (144 of 308 [46.8% (95% CI, 41.2%-52.9%)]) reported that the community pharmacy in which they work provided prescriptions for self-administered hormonal contraception (eg, birth control pills, patch, ring, or injection) (Table). Fewer pharmacists employed by independent pharmacies reported that their pharmacy furnished these prescriptions than those employed by chain pharmacies (51 of 127 [40.2%] vs 93 of 174 [53.4%]; prevalence ratio [PR], 0.75 [95% CI, 0.58-0.97]) (eTable 4 in Supplement 1). Slightly more than three-quarters of participants (243 of 308 [78.9% (95% CI, 74.7%-83.5%)]) reported that the pharmacies where they worked offered levonorgestrel emergency contraception (eg, Plan B One-Step) without an outside clinician’s prescription (ie, over-the-counter or pharmacist prescribed).

### Attitudes Toward Medication Abortion

Most pharmacists (193 of 280 [68.9% (95% CI, 63.1%-74.3%)]) indicated that they would be willing to prescribe medication abortion to pharmacy clients if it were allowed by law (Figure 1 and eTable 2 in Supplement 1). However, slightly less than half (139 of 288 [48.3% (95% CI, 42.4%-54.2%)]) were confident in their knowledge of medication abortion and only 115 of 285 (40.4% [95% CI, 34.6%-46.3%]) were confident in their ability to prescribe medication abortion.

**Figure 2.**
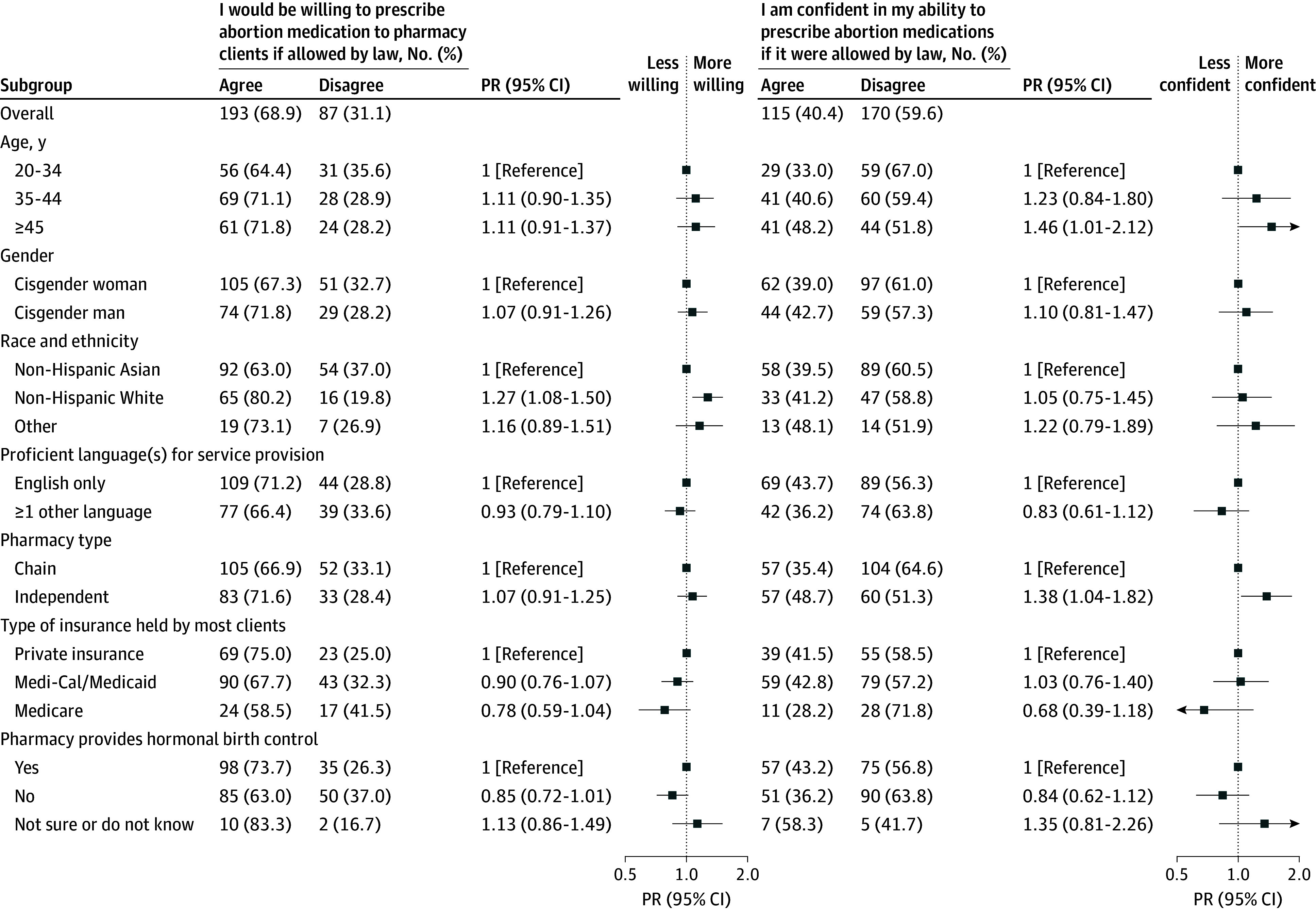
Attitudes About Medication Abortion by Characteristics of Pharmacists and Community Pharmacies in the California Pharmacist Survey, 2022 Prevalence ratios (PRs) are estimated via bivariate log-binomial regression (see eTable 5 in Supplement 1).

**Figure 3.**
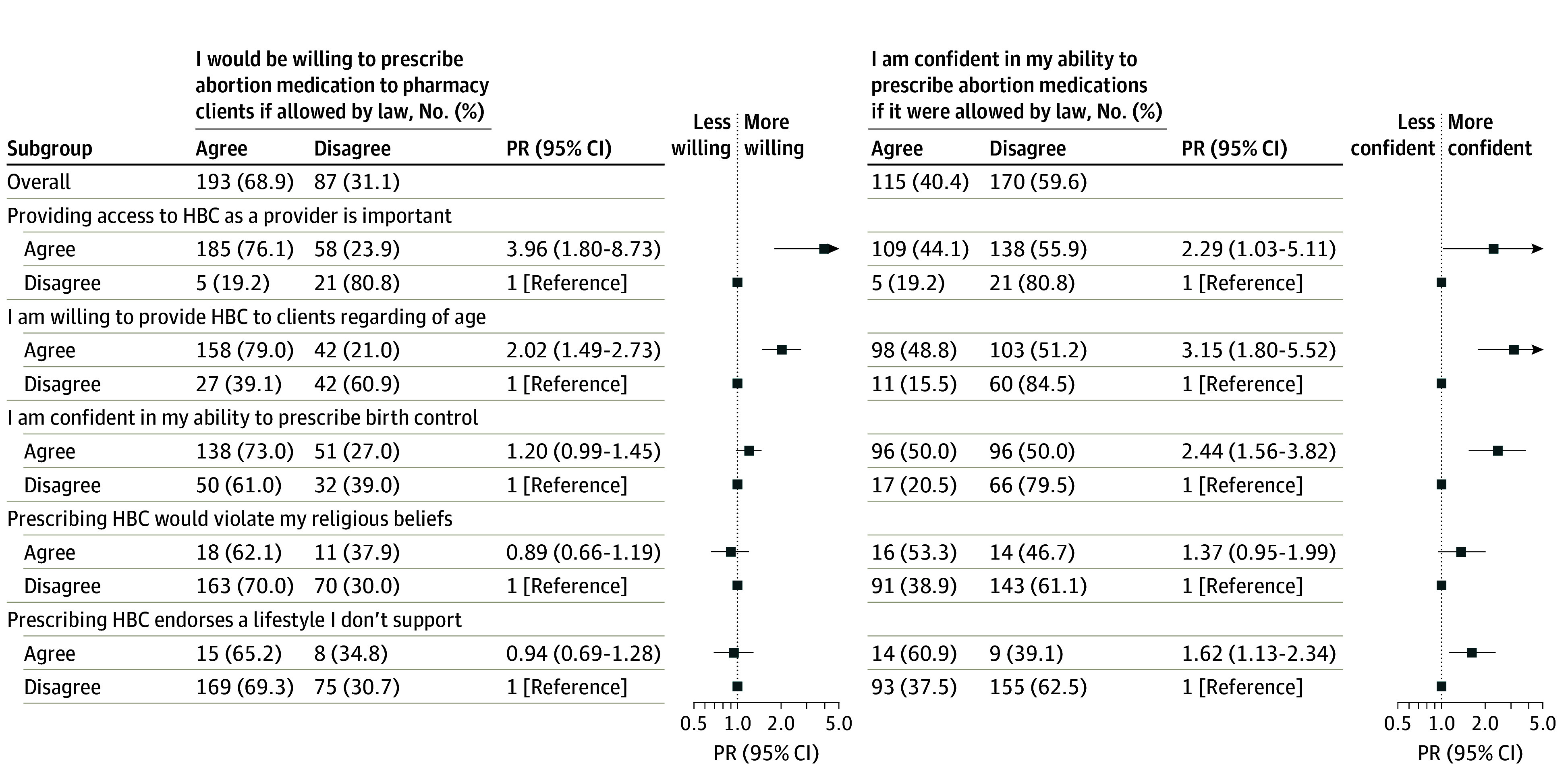
Attitudes About Medication Abortion by Attitudes About Birth Control Among Pharmacists in the California Pharmacist Survey, 2022 Prevalence ratios (PR) are estimated via bivariate log-binomial regression (see eTable 5 in Supplement 1). HBC indicates hormonal birth control.

Associations between pharmacist and pharmacy characteristics and attitudes toward medication abortion are displayed in Figure 2 and Figure 3 (eTable 5 in Supplement 1). A slightly larger proportion of non-Hispanic White pharmacists indicated a willingness to prescribe abortion medication if it were allowed by law than their non-Hispanic Asian peers (PR, 1.27 [95% CI, 1.08-1.50]) (Figure 2). Larger proportions of pharmacists 45 years or older than those aged 20 to 34 years (PR, 1.46 [95% CI, 1.01-2.12]) and those who worked at independent pharmacies than those who worked in chain pharmacies (PR, 1.38 [95% CI, 1.04-1.82]) expressed confidence in their ability to prescribe abortion medications.

As hypothesized, pharmacist attitudes toward hormonal and emergency contraception were positively associated with attitudes toward medication abortion (Figure 3). Pharmacists who agreed that providing access to hormonal contraception as a prescribing provider is important were 3.96 (95% CI, 1.80-8.73) times as likely to indicate willingness to prescribe abortion medication to pharmacy clients if allowed by law than those who disagreed. Those who were confident in their ability to prescribe birth control were 2.44 (95% CI, 1.56-3.82) times as likely to report confidence in their ability to prescribe abortion medication than those who were not. Although few pharmacists (n = 26) agreed that prescribing hormonal birth control would mean endorsing a lifestyle they do not support, those who did were more likely to report confidence in their ability to prescribe abortion medication (PR, 1.62 [95% CI, 1.13-2.34]).

### Perceived Pharmacy-Level Barriers to Pharmacist-Provided Prescriptions for Hormonal Birth Control

Among participants at pharmacies that did not provide self-administered hormonal contraception (n = 149), the most frequently endorsed barriers to doing so were lack of knowledge or training (65 [43.6% (95% CI, 35.6%-51.7%)]), insufficient staff or time to add new services (58 [38.9% (95% CI, 31.5%-47.3%)]), and lack of insurance coverage for service provision (50 [33.6% (95% CI, 26.2%-41.3%)]) (eTable 6 in Supplement 1). Liability concerns (33 [22.1% (95% CI, 16.1%-29.0%)]), difficulty obtaining medical history (28 [18.8% (95% CI, 13.4%-25.4%)]), and not enough demand for the service among clients (27 [18.1% (95% CI, 12.8%-24.6%)]) were reported by some participants. Relatively few participants reported difficulty verifying medical eligibility (12 [8.1% (95% CI, 4.7%-12.6%)]), personal beliefs (10 [6.7% (95% CI, 3.4%-10.5%)]), and other barriers (4 [2.7% (95% CI, 0.7%-5.0%)]). Pharmacists who worked in chain pharmacies more often endorsed not enough staff or time to add services as a barrier to pharmacist-prescribed hormonal birth control than their peers who worked in independent pharmacies (36 of 72 [50.0%] vs 20 of 70 [28.6%]; PR, 1.75 [95% CI, 1.13-2.71]) (eTable 7 in Supplement 1).

## Discussion

Most licensed California pharmacists working at community pharmacies who participated in this study (68.9%) indicated their willingness to prescribe medication abortion if it were allowed by law. However, fewer than half were confident in their knowledge of or ability to prescribe abortion medication. Pharmacists who believed that prescribing hormonal birth control was important were also likely to report that they were willing to provide medication abortion, as hypothesized. Similarly, pharmacists who felt confident in their knowledge of and ability to prescribe self-administered hormonal birth control were also more confident in their knowledge of and ability to prescribe medication abortion. Taken together, these findings suggest that pharmacies may be a feasible channel for the provision of medication abortion.

Despite high levels of pharmacist support for pharmacist-prescribed hormonal birth control observed in this study—consistent with past studies^23^—slightly less than half of licensed, community pharmacists (46.8%) reported that the pharmacy in which they work provided prescriptions for self-administered hormonal contraception (eg, birth control pills, patch, ring, or injection). In this study, we observed that more chain pharmacies offered pharmacist-provided hormonal contraception than independent pharmacies. Prior studies^24,25,26,27^ have similarly found that chain pharmacies are more likely to provide emergency contraception without restrictions (over-the-counter and without security barriers). This suggests that chain pharmacies are more experienced in providing reproductive health care medications directly to clients. Furthermore, the corporate structure behind chain pharmacies may be responsible for reducing barriers, as policies can be set at the corporate level to facilitate access, and greater financial resources could enable stocking of over-the-counter medications.^28^

In this study, we also observed that emergency contraception was far more available than pharmacist-prescribed hormonal birth control; however, nearly one-fifth of community pharmacists indicated that emergency contraception was not available where they worked without an outside clinician’s prescription, despite its availability as an over-the-counter product. This finding is consistent with those from a 2017 secret shopper study conducted in Los Angeles County^29^ that found that over-the-counter emergency contraception was not available at approximately 23% of pharmacies. Recent studies suggest that the availability of over-the-counter emergency contraception may be even more limited elsewhere in the US.^24,28,30,31,32^

California was one of the first states to expand pharmacist scope of practice to include furnishing contraception, which is now permitted in 27 states plus the District of Columbia.^21^ Although California was an early pioneer in this area, implementation lagged behind policy change: 1 year after implementation, only 5.1% of California pharmacists reported furnishing hormonal contraception^33^; 3 years in, 11% of Los Angeles County pharmacies reported implementation.^29^ Levels of implementation have varied between states (eg, 19% in New Mexico 2 years after the change in law, and 31% in Hawaii and 46% in Oregon 3 years post expansion)^34,35^ indicating that significant opportunities to expand access to contraception remain. Across states, consistent with our findings, pharmacists reported barriers to incorporating hormonal contraception into their practice, including training needs, payment for pharmacist services, time and staff constraints, and liability concerns.^23,36,37^

In this study, we observed that slightly fewer Asian pharmacists than White pharmacists indicated a willingness to prescribe abortion medication. Given that the race and ethnicity of pharmacists may be correlated with the demographic composition of pharmacist catchment areas, future efforts to ensure access to reproductive health services should be attentive to area sociodemographic composition. Lessons learned in Oregon suggest that state efforts to support implementation can increase access to contraception beyond the passage of laws and across the state.^38^ Before the law took effect, the state convened a task force to identify potential barriers and to guide implementation. Within 12 months of expanded pharmacist practice, 63% of zip codes in Oregon had a pharmacist who prescribed contraception.

### Strengths and Limitations

A strength of this study is that the involvement of pharmacists in the study design and implementation increases confidence that the data gathered have the potential to inform pharmacy practice. Additionally, the survey was implemented using rigorous, best-practice procedures to ensure data integrity for internet research.

This study also has some limitations. We relied on pharmacist reports of pharmacy practice to ascertain the availability of pharmacist-prescribed hormonal birth control, which renders the study vulnerable to information bias.^39^ Pharmacists who themselves provide a service may be more likely to report that the pharmacy offers the service than those who have not provided the service themselves (but may lack information about store practice). Another consideration is the possibility that pharmacists were clustered within pharmacies, which, if present, could affect tests of statistical significance. Detailed workplace information (ie, name and street address) was not collected. However, 243 of 313 participants (77.6%) who reported a zip code for their pharmacy did not share it with any other participant working in the same type of pharmacy (eg, chain, independent). Thus, our analyses suggest that most participants were the only respondent from their pharmacy.

The use of nonprobability methods to gather data, and the associated risk of selection bias,^39^ is another study limitation. It is possible that our results may not be representative of the attitudes and perspectives of the larger population of licensed community pharmacists in California. However, the age, gender, and racial and ethnic distribution of the sample is similar to that of California pharmacists more broadly (eTable 1 in Supplement 1), and the geographic distribution of participants’ pharmacies mirrors the population distribution of California,^40^ providing some evidence in support of the demographic representativeness of our sample.

## Conclusions

The findings of this cross-sectional survey study suggest that most pharmacists in California would be willing to prescribe medication abortion in the future, were they legally permitted to do so. However, efforts to expand provider scope of practice to increase abortion access would likely need to address moderate levels of confidence in, knowledge of, and ability to prescribe medication abortion. Furthermore, legislative efforts to expand abortion access through an expanded scope of practice for pharmacists should be informed by experience with California law SB 493, which has allowed them to prescribe hormonal birth control since 2016. Although most pharmacists held favorable attitudes toward pharmacist-provided hormonal contraception, just under half of participants (46.8%) report that the community-based pharmacies in which they work offer this service.

Implementation barriers identified through this study, and prior research, including lack of pharmacist knowledge, insufficient staff to add new services, and lack of insurance coverage for service provision, can be addressed through the development of sexual and reproductive health service training plans and expanded insurance payment for pharmacist-provided services. Findings also suggest that pharmacies and pharmacists who are already prescribing birth control are likely to be early adopters of pharmacist-prescribed medication abortion and could be prioritized in any future rollout. Finally, studies that draw large probability samples of community pharmacists and include embedded validation studies (eg, secret shopper, interviews with pharmacy owners or chain managers) are recommended. Such surveys could be part of a system to monitor reproductive health service and product availability in the state.

## Notes

Supplement 1. **eTable 1.** Demographic Comparison of Participants in the California Pharmacist Survey (2022) With External Data (2013-2017) **eTable 2.** Pharmacist Attitudes About Birth Control and Medication Abortion Provision in the California Pharmacist Survey (n = 316), 2022 **eTable 3.** Attitudes About Birth Control and Medication Abortion Provision in the California Pharmacist Survey (N = 919) by Participant Type, 2022 **eTable 4.** Pharmacy Provision of Hormonal Contraception by Characteristics of Pharmacists and Community Pharmacies in the California Pharmacist Survey, 2022 **eTable 5.** Attitudes About Medication Abortion by Characteristics of Pharmacists and Community Pharmacies in the California Pharmacist Survey, 2022 **eTable 6.** Barriers to Hormonal Contraceptive Provision Among Pharmacists Who Reported Working in a Community Pharmacy That Does Not Provide Self-Administered Hormonal Contraception Without an Outside Provider’s Prescription (n = 149), 2022 **eTable 7.** Barriers to Hormonal Contraceptive Provision Among Pharmacists Who Reported Working in a Chain or Independent Community Pharmacy That Does Not Provide Self-Administered Hormonal Contraception Without an Outside Provider’s Prescription by Pharmacy Type (n = 142), 2022

Supplement 2.
Data Sharing Statement
