# Supplementary material for: Willingness of Pharmacists to Prescribe Medication Abortion in California
Source: JAMA Netw Open. 2024 Apr 10;7(4):e246018. doi: 10.1001/jamanetworkopen.2024.6018 (PMC11007579; doi:10.1001/jamanetworkopen.2024.6018)
Supplement: Supplement 1. — eTable 1. Demographic Comparison of Participants in the California Pharmacist Survey (2022) With External Data (2013-2017) eTable 2. Pharmacist Attitudes About Birth Control and Medication Abortion Provision in the California Pharmacist Survey (n = 316), 2022 eTable 3. Attitudes About Birth Control and Medication Abortion Provision in the California Pharmacist Survey (N = 919) by Participant Type, 2022 eTable 4. Pharmacy Provision of Hormonal Contraception by Characteristics of Pharmacists and Community Pharmacies in the California Pharmacist Survey, 2022 eTable 5. Attitudes About Medication Abortion by Characteristics of Pharmacists and Community Pharmacies in the California Pharmacist Survey, 2022 eTable 6. Barriers to Hormonal Contraceptive Provision Among Pharmacists Who Reported Working in a Community Pharmacy That Does Not Provide Self-Administered Hormonal Contraception Without an Outside Provider’s Prescription (n = 149), 2022 eTable 7. Barriers to Hormonal Contraceptive Provision Among Pharmacists Who Reported Working in a Chain or Independent Community Pharmacy That Does Not Provide Self-Administered Hormonal Contraception Without an Outside Provider’s Prescription by Pharmacy Type (n = 142), 2022 [file jamanetwopen-e246018-s001.pdf]

Supplementary Online Content

Cohen C, Hunter L, Beltran R, et al. Willingness of pharmacists to prescribe medication abortion in California. *JAMA Netw Open*. 2024;7(4):e246018. doi:10.1001/jamanetworkopen.2024.6018

eTable 1. Demographic Comparison of Participants in the California Pharmacist Survey (2022) With External Data (2013-2017)
eTable 2. Pharmacist Attitudes About Birth Control and Medication Abortion Provision in the California Pharmacist Survey (n = 316), 2022
eTable 3. Attitudes About Birth Control and Medication Abortion Provision in the California Pharmacist Survey (N = 919) by Participant Type, 2022
eTable 4. Pharmacy Provision of Hormonal Contraception by Characteristics of Pharmacists and Community Pharmacies in the California Pharmacist Survey, 2022
eTable 5. Attitudes About Medication Abortion by Characteristics of Pharmacists and Community Pharmacies in the California Pharmacist Survey, 2022
eTable 6. Barriers to Hormonal Contraceptive Provision Among Pharmacists Who Reported Working in a Community Pharmacy That Does Not Provide Self-Administered Hormonal Contraception Without an Outside Provider's Prescription (n = 149), 2022
eTable 7. Barriers to Hormonal Contraceptive Provision Among Pharmacists Who Reported Working in a Chain or Independent Community Pharmacy That Does Not Provide Self-Administered Hormonal Contraception Without an Outside Provider's Prescription by Pharmacy Type (n = 142), 2022

This supplementary material has been provided by the authors to give readers additional information about their work.

© 2024 Cohen C et al. *JAMA Network Open*.

eTable 1. Demographic Comparison of Participants in the California Pharmacist Survey (2022) With External Data (2013–2017)

	2022 California Pharmacist Survey*		2013–2017 American Communities Survey**
	All practicing pharmacists (n=769)	Practicing community pharmacists (n=316)	California pharmacists
**Age in years (continuous), mean**	39.2	40.9	42.9
**Age in years (categorical), %**			
Under 35	39.9	33.1	33.7
35–44	35.0	35.1	26.1
45–64	20.6	25.0	33.2
65 or older	4.5	6.8	7.0
**Women, %**	63.7	59.3	61.6
**Race and ethnicity, %**			
American Indian or Alaska Native	0.3 (0.3)	0.0 (0.0)	0.03
Asian	63.9 (53.4)	58.5 (50.3)	53.5
Black or African American	2.0 (1.7)	1.8 (1.6)	2.5
Hispanic or Latino	4.7 (3.9)	3.7 (3.2)	5.1
Native Hawaiian or Pacific Islander	0.2 (0.1)	0.4 (0.3)	0.3
White	23.6 (19.8)	30.9 (26.6)	35.5
Multiracial	2.5 (2.1)	2.6 (2.2)	2.7
Other	2.8 (2.3)	2.2 (1.9)	0.3
Unknown or missing	- (16.4)	- (13.9)	-

*Excludes pharmacy students (n=83) and non-practicing pharmacists (n=67). Percentages for age and gender exclude missing and ‘prefer not to specify’ responses from the denominator; percentages for race and ethnicity were calculated excluding and (in parentheses) including missing and ‘prefer not to specify’ responses.
**As reported by the Healthforce Center at UCSF (2020). Percentage shown for Asian race and ethnicity (53.5%) is the sum of Asian (48.4%) and Filipino (5.1%) categories.

© 2024 Cohen C et al. *JAMA Network Open*.

eTable 2. Pharmacist Attitudes About Birth Control and Medication Abortion Provision in the California Pharmacist Survey (n = 316), 2022

Birth control attitudes	n (%*)	%** (95% CI)
**I am confident in my knowledge of hormonal birth control.**		
Strongly agree	59 (19.2)	19.9 (14.5, 25.4)
Agree	190 (61.7)	64.2 (58.8, 69.7)
Disagree	41 (13.3)	13.9 (8.4, 19.3)
Strongly disagree	6 (1.9)	2.0 (0.0, 7.5)
N/A	12 (3.9)	-
**I am confident in my ability to prescribe birth control.**		
Strongly agree	47 (15.3)	16.2 (10.7, 22.3)
Agree	160 (51.9)	55.2 (49.7, 61.3)
Disagree	66 (21.4)	22.8 (17.2, 28.9)
Strongly disagree	17 (5.5)	5.9 (0.3, 12.0)
N/A	18 (5.8)	-
**I am willing to prescribe hormonal birth control to all pharmacy clients, regardless of age.**		
Strongly agree	66 (21.5)	23.0 (17.1, 29.0)
Agree	147 (47.9)	51.2 (45.3, 57.2)
Disagree	56 (18.2)	19.5 (13.6, 25.5)
Strongly disagree	18 (5.9)	6.3 (0.3, 12.3)
N/A	20 (6.5)	-
**I do not want to prescribe hormonal birth control because doing so would violate my religious beliefs.**		
Strongly agree	10 (3.2)	3.6 (0.0, 9.6)
Agree	22 (7.1)	8.0 (2.2, 14.0)
Disagree	82 (26.6)	29.7 (23.9, 35.7)
Strongly disagree	162 (52.6)	58.7 (52.9, 64.7)
N/A	32 (10.4)	-
**Prescribing hormonal birth control would mean that I am endorsing a lifestyle I don’t support.**		
Strongly agree	4 (1.3)	1.4 (0.0, 7.7)
Agree	22 (7.1)	7.7 (2.1, 14.0)
Disagree	100 (32.5)	35.2 (29.6, 41.5)
Strongly disagree	158 (51.3)	55.6 (50.0, 61.9)
N/A	24 (7.8)	-

© 2024 Cohen C et al. *JAMA Network Open*.

Birth control attitudes	n (%*)	%** (95% CI)
**I believe that providing access to hormonal birth control as a prescribing provider is important.**		
Strongly agree	128 (41.7)	44.3 (38.4, 50.5)
Agree	135 (44.0)	46.7 (40.8, 52.9)
Disagree	21 (6.8)	7.3 (1.4, 13.5)
Strongly disagree	5 (1.6)	1.7 (0.0, 8.0)
N/A	18 (5.9)	-
**I believe that providing access to levonorgestrel emergency contraception as a prescribing provider is important.**		
Strongly agree	134 (43.6)	45.6 (39.8, 51.8)
Agree	138 (45.0)	46.9 (41.2, 53.1)
Disagree	16 (5.2)	5.4 (0.0, 11.6)
Strongly disagree	6 (2.0)	2.0 (0.0, 8.2)
N/A	13 (4.2)	-

Medication abortion attitudes	n (%*)	%** (95% CI)
**I am confident in my knowledge of medication abortion.**		
Strongly agree	25 (8.1)	8.7 (2.8, 15.0)
Agree	114 (37.0)	39.6 (33.7, 45.9)
Disagree	112 (36.4)	38.9 (33.0, 45.2)
Strongly disagree	37 (12.0)	12.8 (6.9, 19.2)
N/A	20 (6.5)	-
**I am confident in my ability to prescribe abortion medications if it were allowed by law.**		
Strongly agree	25 (8.1)	8.8 (2.8, 15.1)
Agree	90 (29.2)	31.6 (25.6, 37.9)
Disagree	118 (38.3)	41.4 (35.4, 47.7)
Strongly disagree	52 (16.9)	18.2 (12.3, 24.6)
N/A	23 (7.5)	-
**I would be willing to prescribe abortion medication to pharmacy clients if allowed by law.**		
Strongly agree	73 (23.7)	26.1 (20.0, 32.3)
Agree	120 (39.0)	42.9 (36.8, 49.1)
Disagree	51 (16.6)	18.2 (12.1, 24.4)
Strongly disagree	36 (11.7)	12.9 (6.8, 19.1)
N/A	28 (9.1)	-

CI: confidence interval, N/A: not applicable.
*Percentages excluding missing responses (n~8-9 per question).
**Percentages excluding missing and "N/A" responses.

© 2024 Cohen C et al. *JAMA Network Open*.

eTable 3. Attitudes About Birth Control and Medication Abortion Provision in the California Pharmacist Survey (N = 919) by Participant Type, 2022

Birth control attitudes	Practicing pharmacists n=769 (%)	Pharmacy students n=83 (%)	Non-practicing pharmacists n=67* (%)	Overall N=919 (%)
**I am confident in my knowledge of hormonal birth control.**				
Strongly agree	132 (18.5)	13 (17.8)	7 (11.1)	152 (17.9)
Agree	383 (53.7)	41 (56.2)	37 (58.7)	461 (54.3)
Disagree	168 (23.6)	15 (20.5)	13 (20.6)	196 (23.1)
Strongly disagree	30 (4.2)	4 (5.5)	6 (9.5)	40 (4.7)
**I am confident in my ability to prescribe birth control.**				
Strongly agree	111 (16.0)	12 (20.7)	6 (10.3)	129 (16.0)
Agree	310 (44.8)	26 (44.8)	27 (46.6)	363 (44.9)
Disagree	215 (31.1)	16 (27.6)	18 (31.0)	249 (30.8)
Strongly disagree	56 (8.1)	4 (6.9)	7 (12.1)	67 (8.3)
**I am willing to prescribe hormonal birth control to all pharmacy clients, regardless of age.**				
Strongly agree	171 (25.2)	27 (44.3)	13 (24.1)	211 (26.6)
Agree	325 (47.9)	27 (44.3)	28 (51.9)	380 (47.9)
Disagree	140 (20.6)	6 (9.8)	10 (18.5)	156 (19.7)
Strongly disagree	42 (6.2)	1 (1.6)	3 (5.6)	46 (5.8)
**I do not want to prescribe hormonal birth control because doing so would violate my religious beliefs.**				
Strongly agree	18 (2.7)	2 (3.0)	2 (3.4)	22 (2.8)
Agree	44 (6.6)	5 (7.6)	0 (0.0)	49 (6.2)
Disagree	181 (27.2)	11 (16.7)	18 (31.0)	210 (26.6)
Strongly disagree	423 (63.5)	48 (72.7)	38 (65.5)	509 (64.4)
**Prescribing hormonal birth control would mean that I am endorsing a lifestyle I don't support.**				
Strongly agree	12 (1.8)	2 (2.9)	2 (3.4)	16 (2.0)
Agree	35 (5.1)	5 (7.4)	0 (0.0)	40 (5.0)
Disagree	214 (31.4)	10 (14.7)	21 (35.6)	245 (30.3)
Strongly disagree	420 (61.7)	51 (75.0)	36 (61.0)	507 (62.7)
**I believe that providing access to hormonal birth control as a prescribing provider is important.**				
Strongly agree	342 (48.9)	49 (71.0)	37 (62.7)	428 (51.8)
Agree	301 (43.1)	17 (24.6)	20 (33.9)	338 (40.9)

© 2024 Cohen C et al. *JAMA Network Open*.

Birth control attitudes	Practicing pharmacists n=769 (%)	Pharmacy students n=83 (%)	Non-practicing pharmacists n=67* (%)	Overall N=919 (%)
Disagree	41 (5.9)	0 (0.0)	1 (1.7)	42 (5.1)
Strongly disagree	15 (2.1)	3 (4.3)	1 (1.7)	19 (2.3)
**I believe that providing access to levonorgestrel emergency contraception as a prescribing provider is important.**				
Strongly agree	367 (51.8)	49 (70.0)	36 (60.0)	452 (53.9)
Agree	296 (41.7)	20 (28.6)	20 (33.3)	336 (40.0)
Disagree	35 (4.9)	0 (0.0)	2 (3.3)	37 (4.4)
Strongly disagree	11 (1.6)	1 (1.4)	2 (3.3)	14 (1.7)

Medication abortion attitudes	Practicing pharmacists n=769 (%)	Pharmacy students n=83 (%)	Non-practicing pharmacists n=67* (%)	Overall N=919 (%)
**I am confident in my knowledge of medication abortion.**				
Strongly agree	68 (9.7)	12 (17.6)	5 (8.2)	85 (10.2)
Agree	229 (32.6)	22 (32.4)	29 (47.5)	280 (33.7)
Disagree	301 (42.8)	28 (41.2)	21 (34.4)	350 (42.1)
Strongly disagree	105 (14.9)	6 (8.8)	6 (9.8)	117 (14.1)
**I am confident in my ability to prescribe abortion medications if it were allowed by law.**				
Strongly agree	71 (10.3)	11 (20.0)	6 (9.8)	88 (10.9)
Agree	201 (29.1)	18 (32.7)	25 (41.0)	244 (30.3)
Disagree	290 (42.0)	22 (40.0)	17 (27.9)	329 (40.8)
Strongly disagree	128 (18.6)	4 (7.3)	13 (21.3)	145 (18.0)
**I would be willing to prescribe abortion medication to pharmacy clients if allowed by law.**				
Strongly agree	183 (27.1)	31 (48.4)	17 (28.8)	231 (28.9)
Agree	307 (45.5)	28 (43.8)	30 (50.8)	365 (45.7)
Disagree	111 (16.4)	4 (6.2)	8 (13.6)	123 (15.4)
Strongly disagree	74 (11.0)	1 (1.6)	4 (6.8)	79 (9.9)

N (column %); percentages exclude missing (n~25-27 per question) and “not applicable” responses (n~45-104 per question).

*Includes n=34 retired pharmacists and n=33 other non-practicing pharmacists.

© 2024 Cohen C et al. *JAMA Network Open*.

eTable 4. Pharmacy Provision of Hormonal Contraception by Characteristics of Pharmacists and Community Pharmacies in the California Pharmacist Survey, 2022

	**Participant's pharmacy provides self-administered hormonal contraception without an outside provider's prescription**		
	Yes, n (row %)	No/Don't know, n (row %)	PR (95% CI)
**Overall**	144 (46.8)	164 (53.2)	
**Age in years**			
20-34	49 (50.0)	49 (50.0)	Reference
35-44	54 (51.9)	50 (48.1)	1.04 (0.79, 1.36)
45+	34 (36.2)	60 (63.8)	0.72 (0.52, 1.01)
**Gender**			
Cisgender woman	85 (50.3)	84 (49.7)	Reference
Cisgender man	46 (40.4)	68 (59.6)	0.80 (0.61, 1.05)
**Race and ethnicity**			
Asian, non-Hispanic	73 (45.9)	86 (54.1)	Reference
White, non-Hispanic	37 (44.0)	47 (56.0)	0.96 (0.71, 1.29)
Other race/ethnicity	16 (55.2)	13 (44.8)	1.20 (0.83, 1.74)
**Proficient language(s) for service provision**			
English only	75 (43.9)	96 (56.1)	Reference
At least one other language	62 (49.6)	63 (50.4)	1.13 (0.89, 1.44)
**Proficient language(s) for service provision***			
English only	75 (43.9)	96 (56.1)	Reference
Chinese	16 (39.0)	25 (61.0)	0.89 (0.59, 1.35)
Spanish	20 (54.1)	17 (45.9)	1.23 (0.88, 1.74)
Vietnamese	11 (47.8)	12 (52.2)	1.09 (0.69, 1.73)
Other language(s)	21 (56.8)	16 (43.2)	1.29 (0.93, 1.80)
**Pharmacy type**			
Chain	93 (53.4)	81 (46.6)	Reference
Independent	51 (40.2)	76 (59.8)	0.75 (0.58, 0.97)
**Pharmacy region**			
Los Angeles County	48 (47.1)	54 (52.9)	Reference
San Francisco Bay Area	24 (40.7)	35 (59.3)	0.86 (0.60, 1.25)
Orange County	21 (61.8)	13 (38.2)	1.31 (0.94, 1.84)
Superior California	13 (52.0)	12 (48.0)	1.10 (0.72, 1.70)
Other region	36 (41.9)	50 (58.1)	0.89 (0.64, 1.23)

© 2024 Cohen C et al. *JAMA Network Open*.

	Participant's pharmacy provides self-administered hormonal contraception without an outside provider's prescription		
	Yes, n (row %)	No/Don't know, n (row %)	PR (95% CI)
**Type of insurance held by majority of clients**			
Private insurance	53 (52.0)	49 (48.0)	Reference
Medi-Cal/Medicaid	59 (41.0)	85 (59.0)	0.79 (0.60, 1.03)
Medicare	21 (48.8)	22 (51.2)	0.94 (0.66, 1.34)

PR: prevalence ratio estimated via log-binomial regression, CI: confidence interval.
Excludes missing responses and categories with <10 observations (gender: n=2 "gender-fluid or nonbinary," pharmacy type: n=9 "none of the above," type of insurance: n=7 "uninsured or other insurance").
*Non-referent categories are not mutually exclusive and were compared to referent ("English only") in separate models.

© 2024 Cohen C et al. *JAMA Network Open*.

eTable 5. Attitudes About Medication Abortion by Characteristics of Pharmacists and Community Pharmacies in the California Pharmacist Survey, 2022

	Willing to prescribe abortion medication to pharmacy clients if allowed by law			Confident in knowledge of medication abortion			Confident in ability to prescribe abortion medications if allowed by law		
	Agree, n (row %)	Disagree, n (row %)	PR (95% CI)	Agree, n (row %)	Disagree, n (row %)	PR (95% CI)	Agree, n (row %)	Disagree, n (row %)	PR (95% CI)
**Overall**	193 (68.9)	87 (31.1)		139 (48.3)	149 (51.7)		115 (40.4)	170 (59.6)	
**Age in years**									
20–34	56 (64.4)	31 (35.6)	Reference	39 (43.3)	51 (56.7)	Reference	29 (33.0)	59 (67.0)	Reference
35–44	69 (71.1)	28 (28.9)	1.11 (0.90, 1.35)	47 (47.0)	53 (53.0)	1.08 (0.79, 1.49)	41 (40.6)	60 (59.4)	1.23 (0.84, 1.80)
45+	61 (71.8)	24 (28.2)	1.11 (0.91, 1.37)	46 (52.9)	41 (47.1)	1.22 (0.90, 1.66)	41 (48.2)	44 (51.8)	1.46 (1.01, 2.12)
**Gender**									
Cisgender woman	105 (67.3)	51 (32.7)	Reference	73 (46.2)	85 (53.8)	Reference	62 (39.0)	97 (61.0)	Reference
Cisgender man	74 (71.8)	29 (28.2)	1.07 (0.91, 1.26)	53 (49.5)	54 (50.5)	1.07 (0.83, 1.38)	44 (42.7)	59 (57.3)	1.10 (0.81, 1.47)
**Race and ethnicity**									
Asian, non-Hispanic	92 (63.0)	54 (37.0)	Reference	63 (42.9)	84 (57.1)	Reference	58 (39.5)	89 (60.5)	Reference
White, non-Hispanic	65 (80.2)	16 (19.8)	1.27 (1.08, 1.50)	46 (56.8)	35 (43.2)	1.33 (1.02, 1.73)	33 (41.2)	47 (58.8)	1.05 (0.75, 1.45)
Other race/ethnicity	19 (73.1)	7 (26.9)	1.16 (0.89, 1.51)	13 (46.4)	15 (53.6)	1.08 (0.70, 1.68)	13 (48.1)	14 (51.9)	1.22 (0.79, 1.89)
**Proficient language(s) for service provision**									
English only	109 (71.2)	44 (28.8)	Reference	80 (50.3)	79 (49.7)	Reference	69 (43.7)	89 (56.3)	Reference
At least one other language	77 (66.4)	39 (33.6)	0.93 (0.79, 1.10)	52 (44.1)	66 (55.9)	0.88 (0.68, 1.13)	42 (36.2)	74 (63.8)	0.83 (0.61, 1.12)
**Proficient language(s) for service provision***									
English only	109 (71.2)	44 (28.8)	Reference	80 (50.3)	79 (49.7)	Reference	69 (43.7)	89 (56.3)	Reference
Chinese	26 (66.7)	13 (33.3)	0.94 (0.73, 1.19)	14 (35.9)	25 (64.1)	0.71 (0.46, 1.12)	11 (28.2)	28 (71.8)	0.65 (0.38, 1.10)
Spanish	22 (66.7)	11 (33.3)	0.94 (0.72, 1.22)	20 (55.6)	16 (44.4)	1.10 (0.79, 1.54)	15 (41.7)	21 (58.3)	0.95 (0.62, 1.46)
Vietnamese	14 (66.7)	7 (33.3)	0.94 (0.68, 1.29)	11 (55.0)	9 (45.0)	1.09 (0.71, 1.67)	10 (50.0)	10 (50.0)	1.14 (0.71, 1.84)
Other language(s)	25 (71.4)	10 (28.6)	1.00 (0.79, 1.27)	15 (42.9)	20 (57.1)	0.85 (0.56, 1.29)	13 (38.2)	21 (61.8)	0.88 (0.55, 1.39)

© 2024 Cohen C et al. *JAMA Network Open*.

	Willing to prescribe abortion medication to pharmacy clients if allowed by law			Confident in knowledge of medication abortion			Confident in ability to prescribe abortion medications if allowed by law		
	Agree, n (row %)	Disagree, n (row %)	PR (95% CI)	Agree, n (row %)	Disagree, n (row %)	PR (95% CI)	Agree, n (row %)	Disagree, n (row %)	PR (95% CI)
**"I believe that providing access to hormonal birth control as a prescribing provider is important."**									
Agree	185 (76.1)	58 (23.9)	3.96 (1.80, 8.73)	129 (51.0)	124 (49.0)	1.47 (0.86, 2.53)	109 (44.1)	138 (55.9)	2.29 (1.03, 5.11)
Disagree	5 (19.2)	21 (80.8)	Reference	9 (34.6)	17 (65.4)	Reference	5 (19.2)	21 (80.8)	Reference
**"I believe that providing access to levonorgestrel emergency contraception as a prescribing provider is important."**									
Agree	187 (74.2)	65 (25.8)	2.97 (1.38, 6.36)	129 (49.6)	131 (50.4)	1.56 (0.84, 2.91)	113 (44.3)	142 (55.7)	4.87 (1.29, 18.4)
Disagree	5 (25.0)	15 (75.0)	Reference	7 (31.8)	15 (68.2)	Reference	2 (9.1)	20 (90.9)	Reference
**"I am willing to provide hormonal birth control to all pharmacy clients, regardless of age."**									
Agree	158 (79.0)	42 (21.0)	2.02 (1.49, 2.73)	113 (54.9)	93 (45.1)	1.83 (1.25, 2.67)	98 (48.8)	103 (51.2)	3.15 (1.80, 5.52)
Disagree	27 (39.1)	42 (60.9)	Reference	21 (30.0)	49 (70.0)	Reference	11 (15.5)	60 (84.5)	Reference
**"I am confident in my knowledge of hormonal birth control."**									
Agree	160 (69.6)	70 (30.4)	1.08 (0.85, 1.36)	129 (54.0)	110 (46.0)	3.39 (1.70, 6.76)	101 (43.3)	132 (56.7)	1.85 (1.08, 3.17)
Disagree	29 (64.4)	16 (35.6)	Reference	7 (15.9)	37 (84.1)	Reference	11 (23.4)	36 (76.6)	Reference
**"I am confident in my ability to prescribe birth control."**									
Agree	138 (73.0)	51 (27.0)	1.20 (0.99, 1.45)	113 (57.1)	85 (42.9)	2.17 (1.48, 3.20)	96 (50.0)	96 (50.0)	2.44 (1.56, 3.82)
Disagree	50 (61.0)	32 (39.0)	Reference	21 (26.2)	59 (73.8)	Reference	17 (20.5)	66 (79.5)	Reference

© 2024 Cohen C et al. *JAMA Network Open*.

	Willing to prescribe abortion medication to pharmacy clients if allowed by law			Confident in knowledge of medication abortion			Confident in ability to prescribe abortion medications if allowed by law		
	Agree, n (row %)	Disagree, n (row %)	PR (95% CI)	Agree, n (row %)	Disagree, n (row %)	PR (95% CI)	Agree, n (row %)	Disagree, n (row %)	PR (95% CI)
**"I do not want to prescribe hormonal birth control because doing so would violate my religious beliefs."**									
Agree	18 (62.1)	11 (37.9)	0.89 (0.66, 1.19)	18 (60.0)	12 (40.0)	1.30 (0.94, 1.79)	16 (53.3)	14 (46.7)	1.37 (0.95, 1.99)
Disagree	163 (70.0)	70 (30.0)	Reference	109 (46.2)	127 (53.8)	Reference	91 (38.9)	143 (61.1)	Reference
**"Prescribing hormonal birth control would mean that I am endorsing a lifestyle I don't support."**									
Agree	15 (65.2)	8 (34.8)	0.94 (0.69, 1.28)	15 (65.2)	8 (34.8)	1.44 (1.03, 1.99)	14 (60.9)	9 (39.1)	1.62 (1.13, 2.34)
Disagree	169 (69.3)	75 (30.7)	Reference	114 (45.4)	137 (54.6)	Reference	93 (37.5)	155 (62.5)	Reference
**Pharmacy type**									
Chain	105 (66.9)	52 (33.1)	Reference	73 (44.8)	90 (55.2)	Reference	57 (35.4)	104 (64.6)	Reference
Independent	83 (71.6)	33 (28.4)	1.07 (0.91, 1.25)	65 (55.1)	53 (44.9)	1.23 (0.97, 1.56)	57 (48.7)	60 (51.3)	1.38 (1.04, 1.82)
**Pharmacy region**									
Los Angeles County	64 (71.1)	26 (28.9)	Reference	43 (45.7)	51 (54.3)	Reference	34 (37.4)	57 (62.6)	Reference
San Francisco Bay Area	36 (63.2)	21 (36.8)	0.89 (0.70, 1.13)	25 (44.6)	31 (55.4)	0.98 (0.68, 1.41)	21 (37.5)	35 (62.5)	1.00 (0.65, 1.54)
Orange County	20 (64.5)	11 (35.5)	0.91 (0.68, 1.22)	17 (54.8)	14 (45.2)	1.20 (0.81, 1.77)	14 (43.8)	18 (56.2)	1.17 (0.73, 1.88)
Superior California	15 (71.4)	6 (28.6)	1.00 (0.74, 1.36)	13 (56.5)	10 (43.5)	1.24 (0.81, 1.88)	12 (52.2)	11 (47.8)	1.40 (0.87, 2.24)
Other region	56 (70.9)	23 (29.1)	1.00 (0.82, 1.21)	40 (48.8)	42 (51.2)	1.07 (0.78, 1.46)	34 (41.5)	48 (58.5)	1.11 (0.77, 1.61)
**Type of insurance held by majority of clients**									
Private insurance	69 (75.0)	23 (25.0)	Reference	49 (51.6)	46 (48.4)	Reference	39 (41.5)	55 (58.5)	Reference
Medi-Cal/Medicaid	90 (67.7)	43 (32.3)	0.90 (0.76, 1.07)	65 (46.8)	74 (53.2)	0.91 (0.70, 1.18)	59 (42.8)	79 (57.2)	1.03 (0.76, 1.40)
Medicare	24 (58.5)	17 (41.5)	0.78 (0.59, 1.04)	17 (42.5)	23 (57.5)	0.82 (0.55, 1.24)	11 (28.2)	28 (71.8)	0.68 (0.39, 1.18)

© 2024 Cohen C et al. *JAMA Network Open*.

	Willing to prescribe abortion medication to pharmacy clients if allowed by law			Confident in knowledge of medication abortion			Confident in ability to prescribe abortion medications if allowed by law		
	Agree, n (row %)	Disagree, n (row %)	PR (95% CI)	Agree, n (row %)	Disagree, n (row %)	PR (95% CI)	Agree, n (row %)	Disagree, n (row %)	PR (95% CI)
**Pharmacy provision of hormonal contraception**									
Yes	98 (73.7)	35 (26.3)	Reference	72 (52.9)	64 (47.1)	Reference	57 (43.2)	75 (56.8)	Reference
No	85 (63.0)	50 (37.0)	0.85 (0.72, 1.01)	60 (42.9)	80 (57.1)	0.81 (0.63, 1.04)	51 (36.2)	90 (63.8)	0.84 (0.62, 1.12)
Not sure/Don’t know	10 (83.3)	2 (16.7)	1.13 (0.86, 1.49)	7 (58.3)	5 (41.7)	1.10 (0.67, 1.82)	7 (58.3)	5 (41.7)	1.35 (0.81, 2.26)

PR: prevalence ratio estimated via log-binomial regression, CI: confidence interval.
Excludes missing and “not applicable” responses and categories with <10 observations (gender: n=2 “gender-fluid or nonbinary,” pharmacy type: n=9 “none of the above,” type of insurance: n=7 “uninsured or other insurance”).
*Non-referent categories are not mutually exclusive and were compared to referent (“English only”) in separate models.

© 2024 Cohen C et al. *JAMA Network Open*.

eTable 6. Barriers to Hormonal Contraceptive Provision Among Pharmacists Who Reported Working in a Community Pharmacy That Does Not Provide Self-Administered Hormonal Contraception Without an Outside Provider's Prescription (n = 149), 2022

	n	% (95% CI)
Lack of knowledge and/or training about hormonal contraception	65	43.6 (35.6, 51.7)
Not enough staff/time to add new services	58	38.9 (31.5, 47.3)
No coverage for the service, even if the medication is covered	50	33.6 (26.2, 41.3)
Liability concerns	33	22.1 (16.1, 29.0)
Difficulties in obtaining medical history	28	18.8 (13.4, 25.4)
Not enough demand for hormonal contraception among clients	27	18.1 (12.8, 24.6)
Difficulties in verifying medical eligibility	12	8.1 (4.7, 12.6)
Personal beliefs	10	6.7 (3.4, 10.5)
Other barrier(s)	4	2.7 (0.7, 5.0)
None	7	4.7 (2.0, 7.9)

CI: confidence interval.

eTable 7. Barriers to Hormonal Contraceptive Provision Among Pharmacists Who Reported Working in a Chain or Independent Community Pharmacy That Does Not Provide Self-Administered Hormonal Contraception Without an Outside Provider's Prescription by Pharmacy Type (n = 142), 2022

	Chain n=72 (%)	Independent n=70 (%)
Lack of knowledge and/or training about hormonal contraception	32 (44.4)	31 (44.3)
Not enough staff/time to add new services	36 (50.0)	20 (28.6)
No coverage for the service, even if the medication is covered	21 (29.2)	27 (38.6)
Liability concerns	20 (27.8)	12 (17.1)
Difficulties in obtaining medical history	14 (19.4)	14 (20.0)
Not enough demand for hormonal contraception among clients	10 (13.9)	16 (22.9)
Difficulties in verifying medical eligibility	8 (11.1)	4 (5.7)
Personal beliefs	6 (8.3)	3 (4.3)
Other barrier(s)	1 (1.4)	3 (4.3)
None	4 (5.6)	2 (2.9)

N (column %; barriers not mutually exclusive).
Excludes n=7 with community pharmacy type "none of the above."

© 2024 Cohen C et al. *JAMA Network Open*.
