# Supplementary material for: Willingness of Pharmacists to Prescribe Medication Abortion in California
Source: JAMA Netw Open. 2024 Apr 10;7(4):e246018. doi: 10.1001/jamanetworkopen.2024.6018 (PMC11007579; doi:10.1001/jamanetworkopen.2024.6018)
Supplement: Supplement 2. — Data Sharing Statement [file jamanetwopen-e246018-s002.pdf]

Data Sharing Statement

Cohen. Willingness of Pharmacists to Prescribe Medication Abortion in California. *JAMA Netw Open*. Published April 10, 2024. doi:10.1001/jamanetworkopen.2024.6018

Data
Data available: Yes
Data types: Deidentified participant data
How to access data: smccoy@berkeley.edu
When available: With publication

Supporting Documents
Document types: Information sheet and survey
How to access documents: smccoy@berkeley.edu
When available: With publication

Additional Information
Who can access the data: Data are available upon reasonable request.
Types of analyses: Data are available upon reasonable request.
Mechanisms of data availability: With a signed data access agreement.
